# The involvement of FATP1 regulating skeletal muscle fat deposition in stressed broilers was affected by fatty acid substrates

**DOI:** 10.3389/fvets.2022.965894

**Published:** 2022-07-15

**Authors:** Minghui Wang, Hongchao Jiao, Jingpeng Zhao, Hai Lin, Xiaojuan Wang

**Affiliations:** Shandong Provincial Key Laboratory of Animal Biotechnology and Disease Control and Prevention, Department of Animal Science & Technology, Shandong Agricultural University, Taian, China

**Keywords:** broiler chicken, stress, FATP1, fat deposition, skeletal muscle

## Abstract

Fatty acid transport protein 1 (FATP1), plays a major role in the transport and uptake of fatty acids into cells. The effect of FATP1 on the regulation of skeletal muscle fat uptake and deposition in stressed broiler chickens was investigated both *in vivo* and *in vitro*, and the effect of different fatty acid substrates were also included. Dexamethasone (DEX), a synthetic glucocorticoid (GCs), was employed to induce a hyper glucocorticoid milieu and simulate stress. The *in vivo* results showed that DEX would increase the mRNA expression of FATP1 and fat deposition in muscle tissues (*P* < 0.05), the very-low-density lipoprotein (VLDL) and insulin (INS) levels were significantly increased in the plasma by DEX (*P* < 0.05), and the mRNA levels of the glucocorticoid receptor (*GR*), adiponectin receptor (*ADPNR*) and peroxisomal proliferator-activated receptor α (*PPAR*α) in thigh were also up-regulated by DEX (*P* < 0.05). *In vitro* experiment, DEX did not affect the myoblast fat deposition and *PPAR*α and *FATP1* expressions without the external fatty acid (*P* > 0.05). Under PA pre-treatment, both myoblast fatty acid uptake and fat deposition were promoted by DEX treatment (*P* < 0.05), and the effects of DEX on the gene expressions of *GR, ADPNR, PPAR*α and *FATP1* were upregulated first and then downregulated as the dose of DEX increases; while under OA pre-treatment, the myoblast fat deposition was not affected by DEX (*P* > 0.05), the fatty acid uptake was decreased by DEX at 500 nM compared to control (*P* < 0.05). When GR and PPARα were, respectively inhibited by specific inhibitors RU486 and GW6471, the effects of DEX on fatty acid uptake were reversed for PA pre-treated myoblasts (*P* < 0.05) but not for OA pre-treated myoblasts (*P* > 0.05). These results indicate that FATP1 regulation by GCs was affected by fatty acid substrate - saturated fatty acids were favorable for fat uptake and deposition, while unsaturated fatty acids were not. GCs may affect the ADPNR-PPARα-FATP1 pathway by binding to its receptors, thus regulating the uptake of saturated fatty acids into myoblasts.

## Introduction

The modern species of broiler chickens have become increasingly sensitive to stress due to the high demand for their growth rate and feed efficiency (1). Various stressors exist in the intensive rearing of poultry, such as high temperature and limited living space, resulting in stress responses, reducing the production performance and feed efficiency, and causing feed waste (2). Glucocorticoid (GCs) level in the plasma was detected to increase under stress, GCs were thus widely used to establish the stress response model in many studies (2, 3). When stress occurs, GCs is stimulated and released by the activation of the hypothalamic-pituitary-adrenal axis. GCs-regulated energy metabolism plays a vital role in energy mobilization and reallocation to keep the internal environment balance (2–6). Skeletal muscle balances the energy metabolism through the uptake, utilization and storage of energy substrates such as glucose, lipids and amino acids (7, 8). An important metabolic feature of skeletal muscle is the ability to select different energy substrates according to different nutritional environments. Stress leads to ectopic fat storage around and within non-adipose tissues, such as skeletal muscle, enabling energy redistribution (9, 10). Ectopic deposition of fat in the skeletal muscle of poultry reduces feed conversion efficiency, causing energy waste and affecting meat quality (11). In addition, as a major insulin target tissues, muscles ectopic fat accumulation is a key determinant of various related metabolic diseases (12).

Fatty acids are the essential energy resources and structural components for body. The transport, uptake and oxidation of fatty acids are vital for the energy balance of the organism. In the past, scholars believed that free fatty acids were a kind of lipids, similar to the cell membrane in components (13), and they could get through membranes by free diffusion and participate in the intracellular physiological and biochemical processes (14). As the research progressed, many proteins were found to mediate the cross-membrane transport of fatty acids. These proteins could be activated by proteins relevant to insulin pathways (15). Among them, fatty acid transport protein 1 (FATP1) was the first-found member of the solute carrier family and one of the most important proteins. FATP1 could combine specific fatty acids (especially those with long and super-long chains) and carry them through cell membranes (16). Studies revealed that FATP1 played an irreplaceable role in the fat metabolism and even regarded FATP1 as an important target for the treatment of metabolic diseases such as obesity and Type-II diabetes (17–19). Transient FATP1 overexpression in soleus muscle increased palmitate transport by 24% and increased fatty acid oxidation by 35% (20). Electroporation-mediated FATP1 overexpression enhanced palmitate oxidation to CO_2_ (21). Therefore, we hypothesize that GCs may regulate fat deposition in skeletal muscle of stressed broilers through FATP1.

The present study aimed to explore the mechanism by which FATP1 regulates skeletal muscle fat deposition in stressed broiler chickens, the effect of different fatty acid substrates were also included. This research will provide theoretical guidance for exploring the endocrine regulation mechanism responsible for the stressed poultry skeletal muscle development, and help to address the reduction in the production performance of stressed poultry *via* nutritional regulation.

## Materials and methods

### Ethics statement

The animal experiment was reviewed and approved by the Institutional Animal Care and Use Committee of Shandong Agricultural University (No. 2001002) and performed in accordance with the “Guidelines for Experimental Animals” of the Ministry of Science and Technology (Beijing, CN).

### Treatments

#### Animals and *in vivo* treatment

One-day-old male broiler chicks (Arbor Acres, *Gallus gallus domesticus*) were obtained from the Dabao hatchery (Taian, CN). The chicks were reared in an environmentally controlled room. The rearing temperature was maintained at 35°C (65% relative humidity) for the first 2 days and was then gradually reduced to 21°C on day 28 (45% relative humidity) and maintained at 21°C until the end of the experiment. Ninety 35-day-old broilers with similar body weight were randomly divided into three groups: DEX group were injected subcutaneously with dexamethasone (Lukang Cisen Pharmaceutical Corp., Shandong, CN), a synthetic GCs, with the dose of 2.0 mg/kg body weight per day; the Control group was sham-treated with vehicle (0.9% saline); the Pair-fed group was sham-treated with vehicle (0.9% saline) and provided the same amount feed as that consumed by the DEX-treated chickens during the previous day. Each group had 3 pens of 10 chicks. Body weight was recorded daily. The composition and nutrition level of the experimental diet were listed in Table 1.

**Table 1:** The composition and nutrition level of the experimental diet (1–42 days).

**Ingredients**	**1–21 days**	**22–42 days**
Corn (8.0%)	57.5	61.7
Soybean oil	2.2	2.3
Soybean meal, (43% CP)	34.3	29.9
Fish meal (CP >62%)	3.0	3.0
NaCl	0.2	0.2
Limestone	1.2	1.1
CaHPO4	1.0	1.1
Choline	0.3	0.3
Methionine (98%)	0.1	0.2
Mineral premix^a^	0.2	0.2
Vitamin premix^b^	0.1	0.1
**Calculated nutrient composition**		
Crude protein %	21.5	20
Metabolic energy MJ/kg	12.5	12.8
Calcium %	0.8	0.8
Available phosphorus %	0.4	0.4
Lysine %	1.0	0.9
Methionine %	0.4	0.4
Met+Cys, %	0.7	0.7

a *Mineral premix provides the following per kg of diet: Fe (as ferrous sulfate), 80 mg; Zn (as zinc sulfate), 75 mg; Mn (as manganese sulfate), 80 mg; Cu (as copper sulfate) 10 mg, I (as potassium iodide), 0.40 mg; and Se (as sodium selenite), 0.30 mg*.

b *Vitamin premix provides the following per kg of diet: VA, 8,000 IU; VD3, 3,000 IU; VE, 20 IU; VK, 2mg; VB1, 4mg; riboflavin, 8 mg; D-pantothenic acid, 11 mg; VB5, 40 mg; VB6, 4 mg; VB12, 0.02 mg; biotin, 0.15 mg; folic acid, 1.0 mg; choline, 700 mg*.

Before samples were obtained at 38 days of age, chickens were fasted for 12 h. Ten chickens in each group were selected and blood was collected from wing veins. After centrifugation at 3,000 × g for 10 min at 4°C, the plasma samples were obtained and stored at −20°C for further study. The broilers were killed by exsanguination after cervical dislocation. Abdominal fat and livers were harvested and weighted. Samples of breast muscle, thigh muscle, abdominal fat and liver were collected and snap-frozen in liquid nitrogen and stored at −80°C for future analysis.

#### Cells and *in vitro* treatment

Primary cultures of chicken fetal myoblasts were prepared using a modified method described previously by Yablonka-Reuveni and Nameroff (22) and Wang et al. (23). In brief, fertilized eggs were purchased from a commercial source and myoblast cells were isolated from the breast muscle tissues of 16-day-old embryos. The isolated myoblasts were cultured in Dulbecco's modified Eagle's medium (DMEM; HyClone, Thermo Fisher, Shanghai, CN) supplemented with 10% fetal bovine serum and 1% penicillin/streptomycin (Solarbio, Beijing, CN) in a humidified 5% CO_2_ atmosphere at 37°C until the cells reached ~95% confluence.

The isolated myoblasts were cultured in the basal medium (No FA) or medium supplemented with 300 μM oleic acid (OA) or 300 μM palmitic acid (PA) until the following treatments.

##### DEX treatment

Myoblasts were pre-incubated in serum-free medium for 2 h prior to exposure to DEX treatment of 500, 1,000, 2,000, 4,000 nM or no DEX (Control). After a 24-h exposure, the cells were rinsed with D-Hanks' solution, collected and subjected to the further analysis.

##### DEX and GR inhibitors treatment

Myoblasts were pre-incubated in serum-free medium for 2 h prior to exposure to following treatments: Control; DEX (1,000 nM DEX); RU486 (100 nM RU486); DEX+RU486 (1,000 nM DEX and 100 nM RU486). After a 24-h exposure, the cells were rinsed with D-Hanks' solution, collected and subjected to the further analysis.

##### Dexamethasone and PPAR inhibitors treatment

Myoblasts were pre-incubated in serum-free medium for 2 h prior to exposure to following treatments: Control; DEX (1,000 nM DEX); GW6471 (0.5 μM GW6471); DEX+GW6471 (1,000 nM DEX and 0.5 μM GW6471). After 24 h exposure, the cells were rinsed with D-Hanks' solution, collected and subjected to the further analysis.

### Measurements

#### Plasma and intracellular parameters

The glucose (GLU), triglyceride (TG) and insulin (INS) concentrations in plasma were determined using the commercial kits (Jiancheng Biology Engineering Institute, Jiangsu, CN). The concentration of very-low-density lipoprotein (VLDL) was determined using the method described by Griffin and Whitehead (24).

#### Real-time quantitative PCR

The total RNA was extracted from cells or tissues using a total RNA Kit (OMEGA, CT, USA). The quantity and quality of the isolated RNA were measured using a spectrophotometer (Shimadzu Corporation, Kyoto, Japan). Total RNA (1 μg) was reverse-transcribed into first-strand cDNA using Prime ScriptTM RT Master Mix (Takara, Beijing, CN) following the manufacturer's instructions, and quantitative real-time RT-PCR was performed with a SYBR Green I master mix (Takara, Beijing, CN) on ABI 7500 Real-Time PCR System (ABI, CA, USA) at 95°C for 30 s of pre-denaturation, followed by 40 cycles consisting of denaturation at 95°C for 5 s and annealing and extension at 60°C for 34 s. Primers were designed using the Primer 5.0 software (SPS Inc., CA, USA) and were synthesized by Sangon Company (Shanghai, CN). GAPDH (glyceraldehyde phosphate dehydrogenase) and β-Actin was used as internal controls for normalization. The primer sequences are listed in Table 2. The expression levels were quantified using the comparative CT method (2^−Δ*ΔCT*^).

**Table 2:** Gene-specific primer of related genes.

**Gene name**	**Genbank number**	**Primers position**	**Primers sequences(5** **′** ** → 3** **′** **)**	**Product size (bp)**
*ADPN*	NM_206991	Forward	ACCCAGACACAGATGACCGTT	239
		Reverse	GAGCAAGAGCAGAGGTAGGAGT	
*ADPNR1*	NM_001031027	Forward	GGAGAAGGTTGTGTTTGGGATGT	218
		Reverse	TGGAGAGGTAGATGAGTCTTGGC	
*ADPNR2*	NM_001007854	Forward	ACACACAGAGACTGGCAACATC	144
		Reverse	CCCAAGAAGAACAATCCAACAACC	
*GR*	DQ227738	Forward	CATGAACCTCGAAGCTCGCAAGA	126
		Reverse	ACCTCCAGCAGTGACACCAG	
*PPARα*	AF163809	Forward	AGACACCCTTTCACCAGCATCC	167
		Reverse	AACCCTTACAACCTTCACAAGCA	
*FATP1*	DQ352834	Forward	TCAGGAGATGTGTTGGTGATGGAT	138
		Reverse	CGTCTGGTTGAGGATGTGACTC	
*GAPDH*	NM_204305	Forward	CTACACACGGACACTTCAAG	244
		Reverse	ACAAACATGGGGGCATCAG	
*β-Actin*	NM_205518.1	Forward	CTGGCACCTAGCACAATGAA	148
		Reverse	CTGCTTGCTGATCCACATCT	

#### Cell viability

The CCK-8 assay was performed to investigate the viability of myoblasts cultured in 96-well plates. Briefly, 100 μl of CCK-8 (TransGen Biotech, Beijing, CN) solution at a 1:10 dilution was added to each well of the plate, and myoblasts were incubated at 37°C for 2.5 h. Absorbance was measured at 450 nm with a microplate reader (Bio-Tek, Winooski, VT). The mean optical density (OD) of six wells for each indicated group was used to calculate the cell viability percentage.

#### Fatty acid uptake

Fatty acid uptake was assessed by the uptake of fluorescently labeled fatty acids using the QBT Fatty Acid Uptake Assay Kit (R8132, Molecular Devices, CA, USA), according to the manufacturer's instructions. After the experimental treatment, all culture medium was removed from each well and replaced with culture medium in the absence (negative control) or presence of fluorescent fatty acids (final concentration of 300 μg/ml) and incubated at 37°C with 5% CO_2_ for 10 min before flow cytometry analysis. The uptake reaction was stopped by removing the incubation medium and washing the cells twice with pre-cold 1 × PBS (Solarbio, Beijing, CN). Cells were collected by centrifugation at 3,000 rpm for 5 min and subsequently resuspended in 400 μl pre-cold 1 × PBS and maintained at 4°C for later flow cytometry analysis performed within 30 min. For each measurement, data from 10,000 single-cell events were collected using a BD LSRFortessa flow cytometer (BD Biosciences, CA, USA), and each flow cytometric measurement was digitized as fluorescence intensity which was corrected by the negative control.

### Statistical analysis

Data are presented as mean ± SEM. Statistical analysis of the data was performed by one-way ANOVA and SAS statistical software (SAS version 8e, SAS Institute, NC, USA). *P* < 0.05 was considered statistically significant.

## Results

### Effects of DEX on broiler performance

As shown in Table 3, the final body weight and daily weight gain of DEX group were significantly lower than Control and Pair-fed groups (*P* < 0.05). On the contrary, the proportion of the livers and abdominal fat were all significantly increased in the DEX group compared to Pair-fed (*P* < 0.05).

**Table 3:** Effects of dexamethasone on broiler performance.

	**Control**	**DEX**	**Pair-fed**	***P*** **-value**
Body weight (g)	2,037 ± 23.05^a^	1,562 ± 23.26^b^	2,012 ± 21.10^a^	<0.0001
Body weight gain (g/d)	68.92 ± 2.97^a^	−48.27 ± 6.90^c^	50.00 ± 4.98^b^	<0.0001
Liver index (%BW)	2.26 ± 0.12^b^	4.15 ± 0.25^a^	2.12 ± 0.07^b^	<0.0001
Abdominal fat index (%BW)	2.17 ± 0.25^ab^	2.33 ± 0.17^a^	1.66 ± 0.19^b^	0.0819

*Data are presented as mean ± SEM (n = 10). In the same row, different superscripts mean significant difference (P <0.05)*.

### Effects of DEX on blood parameters of broilers

**Figure 1:**
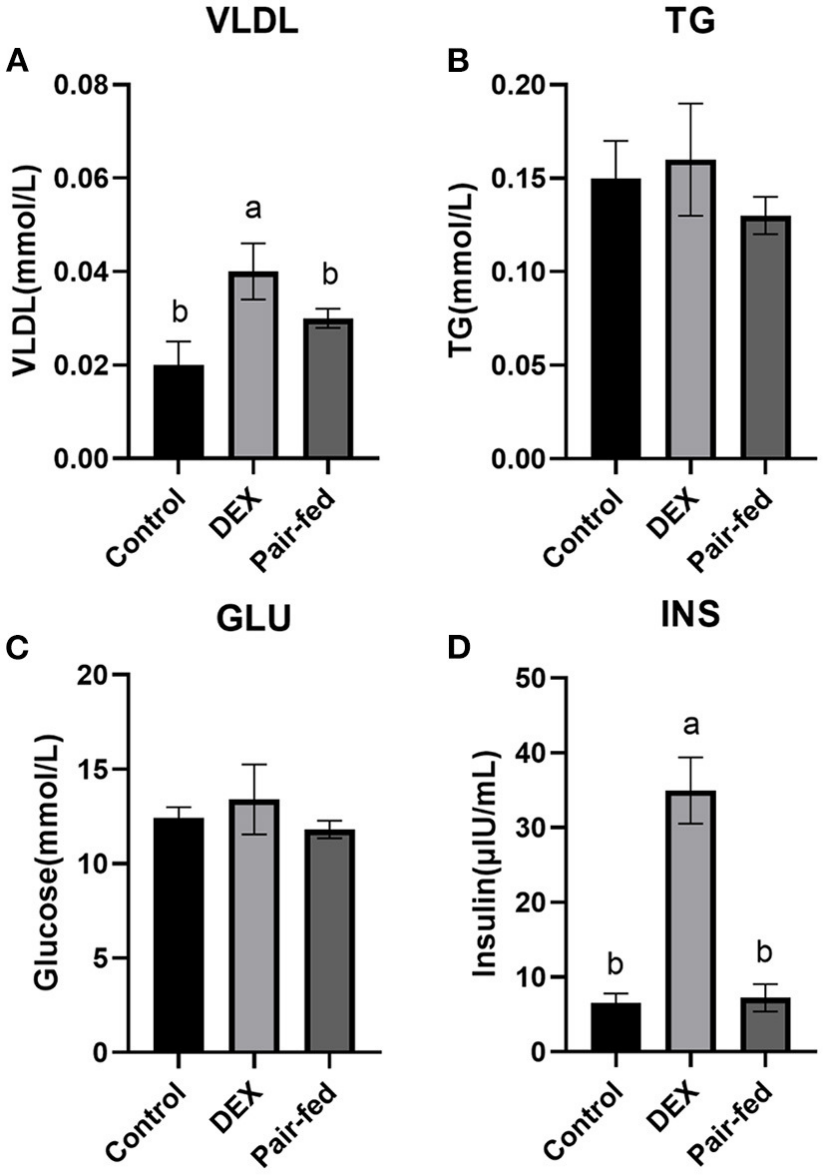
Effects of dexamethasone on blood parameters of broilers. **(A)** VLDL concentration; **(B)** TG concentration; **(C)** GLU concentration; **(D)** INS concentration. Data are presented as the mean ± SEM (*n* = 10). Different superscripts without common letters differ significantly (*P* < 0.05).

The plasma concentrations of VLDL and INS were significantly increased in DEX group than Control and Pair-fed groups (*P* < 0.05, Figures 1A,D). DEX treatment had no significant effect on plasma concentrations of TG and GLU content of broilers (*P* > 0.05, Figures 1B,C).

### Effects of DEX on TG content and gene expressions in tissues

**Figure 2:**
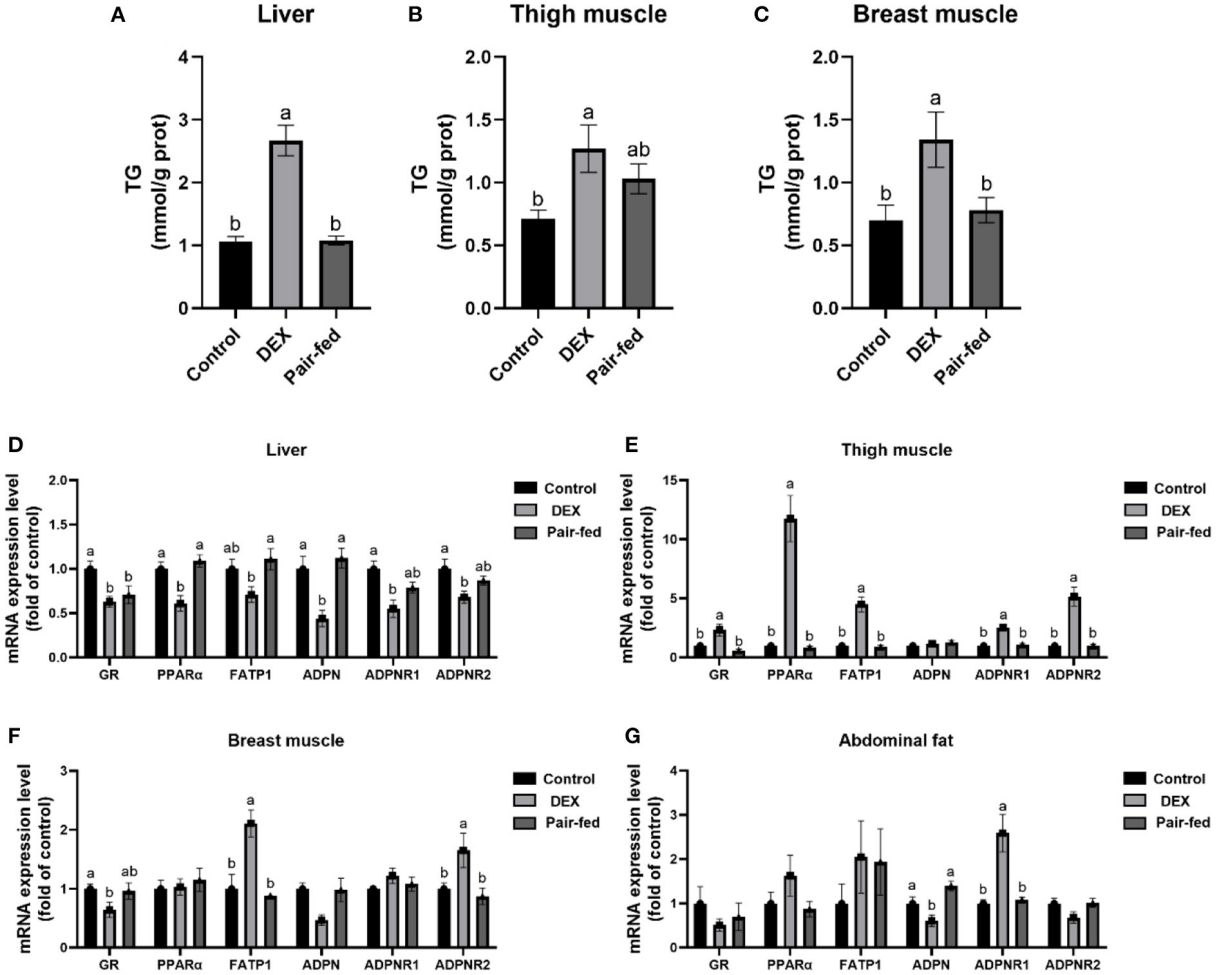
Effects of dexamethasone on TG content and gene expressions in tissues. TG content in liver **(A)**, thigh muscle **(B)**, and breast muscle **(C)**. The mRNA expressions in liver **(D)**, thigh muscle **(E)**, breast muscle **(F)**, and abdominal fat **(G)**. Data are presented as the mean ± SEM (*n* = 10). Different superscripts without common letters differ significantly (*P* < 0.05).

Figure 2 shows that TG content in liver and breast muscle was significantly increased by DEX treatment compared to Control and Pair-fed, and TG content in thigh was significantly increased by DEX treatment compared to Control (*P* < 0.05, Figures 2A–C). The mRNA levels of glucocorticoid receptors (*GR*), peroxisome proliferator-activated receptor α (*PPAR*α), FATP1, adiponectin (*ADPN*), adiponectin receptor 1 (*ADPNR1*), and adiponectin receptor 2 (*ADPNR2*) in livers were significantly down-regulated in DEX group (*P* < 0.05, Figure 2D). The mRNA levels of *GR, PPAR*α, *FATP1, ADPNR1* and *ADPNR2* in thigh muscle and *FATP1* and *ADPNR2* in breast muscle were all significantly up-regulated by DEX compared to the other groups (*P* < 0.05, Figures 2E,F), mRNA expression of GR in breast muscle showed significant decrease (*P* < 0.05, Figure 2F). The mRNA level of *ADPN* was decreased while *ADPNR1* was increased significantly in abdominal fat by DEX compared to Control and Pair-fed (*P* < 0.05, Figure 2G). Other genes presented in Figure 2 were not affected by DEX treatment (*P* > 0.05, Figures 2E–G).

### Effects of DEX on cell viability, TG content and fatty acid uptake in myoblasts cultured with different types of fatty acids or without FA

**Figure 3:**
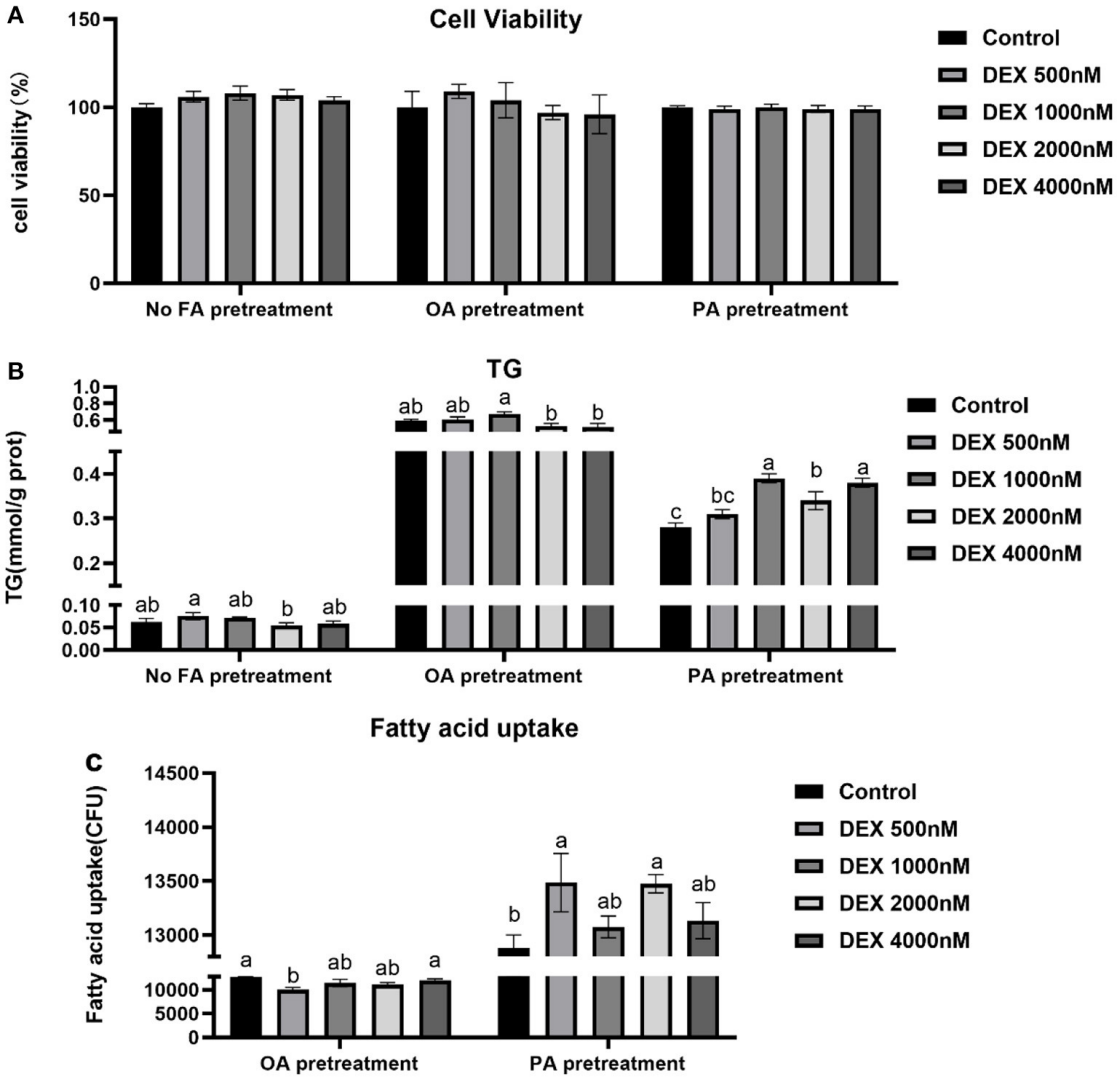
Effects of dexamethasone on cell viability, triglyceride content, and fatty acid uptake in myoblasts pre-treated with different types of fatty acids or no FA pre-treatment. **(A)** Cell viability; **(B)** TG content; **(C)** Fatty acid uptake. Data are presented as the mean ± SEM (*n* = 6). Different superscripts without common letters differ significantly (*P* < 0.05).

Compared to Control, the cell viability and TG content were not significantly different in DEX treated myoblasts without FA pre-treated (*P* > 0.05, Figures 3A,B). DEX didn't affect cell viability in either OA or PA pre-treatment myoblasts (*P* > 0.05, Figure 3A). Compared to Control, the TG content of myoblasts cultured in the PA was increased by DEX which was particularly significant at 1,000 and 4,000 nM (*P* < 0.05, Figure 3B) while not changed by DEX for myoblasts cultured in the OA (*P* > 0.05, Figure 3B). In the myoblasts pre-treated with PA, the fatty acid uptake was increased by DEX compared to Control, especially at 500 and 2,000 nM DEX (*P* < 0.05, Figure 3C), while for myoblasts pre-treated with OA, the fatty acid uptake was decreased by 500 nM DEX (*P* < 0.05) but not changed at other dose of DEX compared to Control (*P* > 0.05, Figure 3C).

### Effects of DEX on gene expressions of myoblasts cultured with different types of fatty acids or without FA

**Figure 4:**
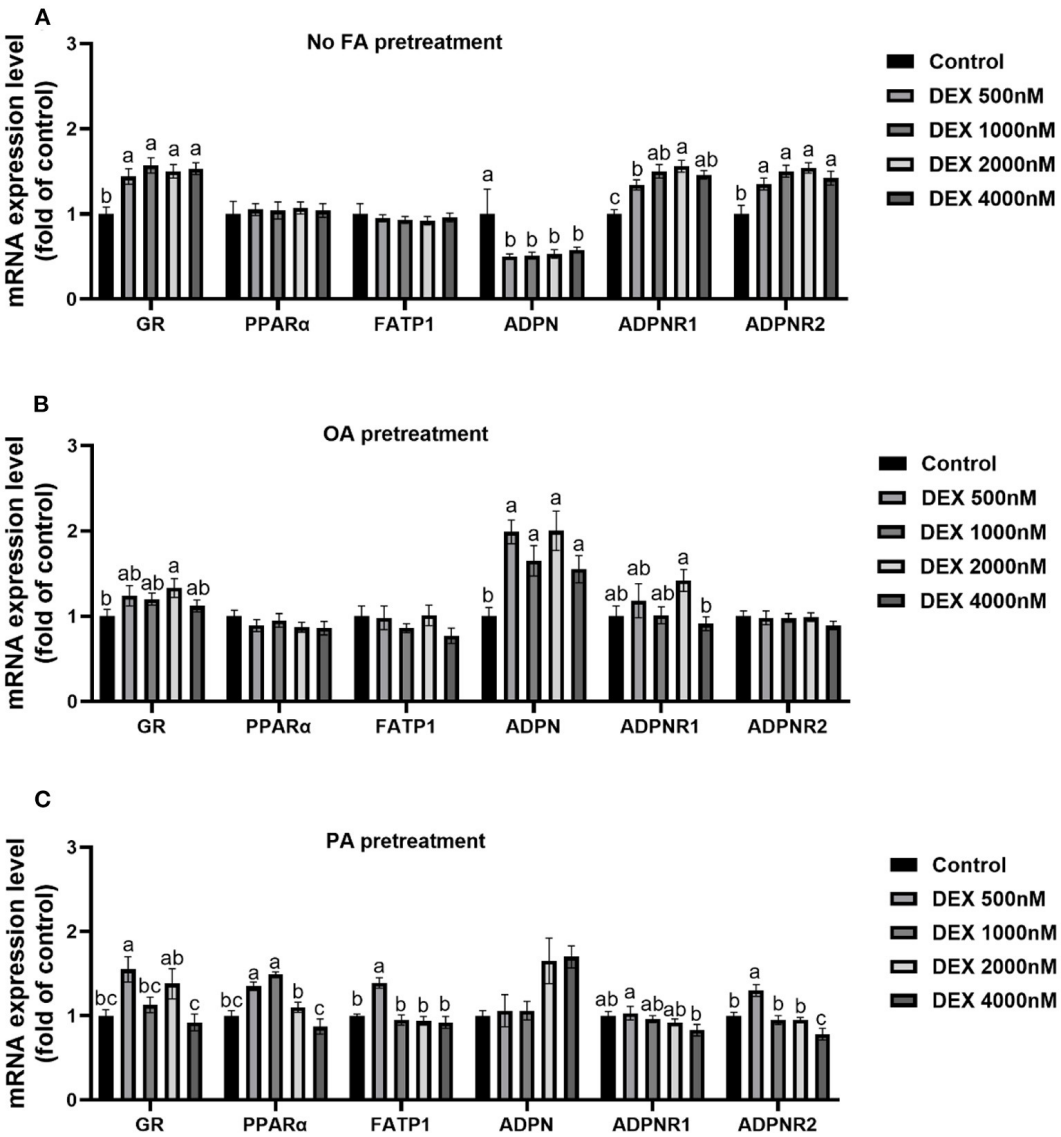
Effects of dexamethasone on mRNA expressions of myoblasts pre-treated with different types of fatty acids or no FA pre-treatment. **(A)** Effects of dexamethasone on mRNA expression level of myoblasts without FA pre-treatment; **(B)** mRNA expression level in myoblasts cultured with OA supplementation medium; **(C)** mRNA expression level in myoblasts cultured with PA supplementation medium. Data are presented as the mean ± SEM (*n* = 6). Different superscripts without common letters differ significantly (*P* < 0.05).

In myoblasts without FA pre-treatment, the mRNA expressions of *GR, ADPNR1* and *ADPNR2* were increased while *ADPN* mRNA expression were decreased significantly by DEX compared to Control (*P* < 0.05, Figure 4A), but *PPAR*α and *FATP1* mRNA expression didn't change between groups (*P* > 0.05, Figure 4A). For myoblasts cultured within the OA medium, *GR* mRNA expression was significantly increased by 2,000 nM DEX and *ADPN* mRNA expression was significantly increased in all DEX groups compared to Control (*P* < 0.05, Figure 4B). For myoblasts cultured within the PA medium, the mRNA levels of *GR, PPAR*α, *FATP1* and *ADPNR2* were up-regulated in lower dose of DEX groups compared to Control (*P* < 0.05, Figure 4C).

### Effects of DEX and inhibitors of GR and PPAR on fatty acid uptake in myoblasts cultured with different types of fatty acids

**Figure 5:**
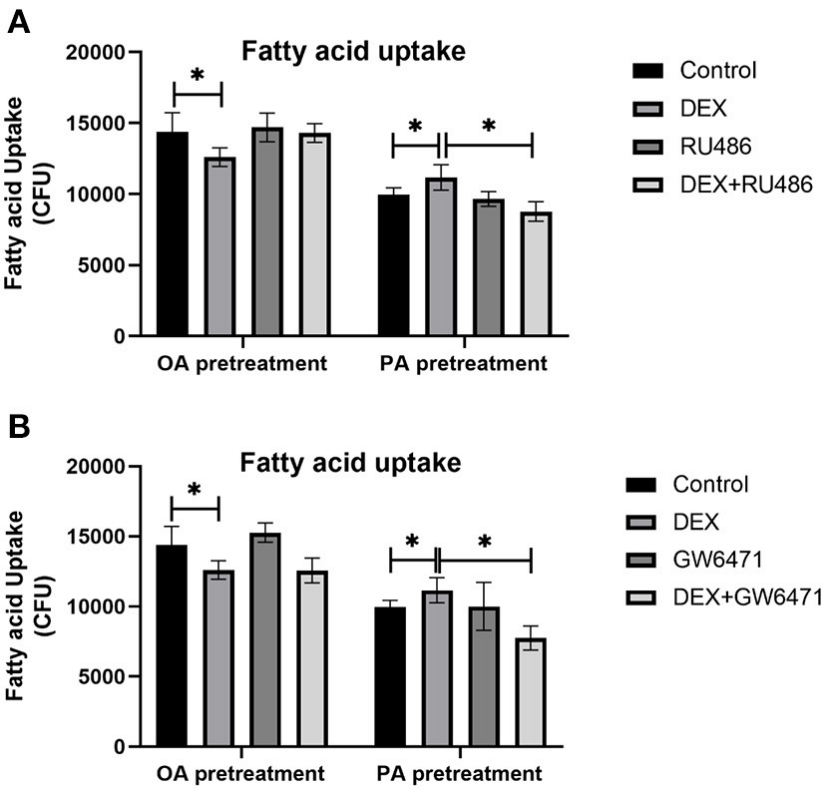
Effects of GR and PPAR inhibitor on fatty acid uptake of myoblasts. **(A)** Effects of GR inhibitor on fatty acid uptake in myoblasts; **(B)** Effects of PPAR inhibitor on fatty acid uptake in myoblasts. Data are presented as the mean ± SEM (*n* = 6). *P < 0.05.

Myoblasts were treated with GR inhibitor (RU486) and PPAR antagonist (GW6471) respectively, to confirm the role of GR and PPAR in fatty acids uptake regulated by DEX. Compared to Control, DEX treatment respectively decreased and increased the fatty acid uptake under OA pre-treatment and PA pre-treatment (*P* < 0.05, Figures 5A,B). For myoblasts with PA pre-treatment, the combined treatment of DEX and RU486 significantly decreased the fatty acid uptake compared with the DEX treatment only (*P* < 0.05, Figure 5A), while for myoblasts with OA pre-treatment, the combined treatment of DEX and RU486 had no effect on fatty acid uptake compared with the DEX treatment only (*P* > 0.05, Figure 5A). In PA pre-treated myoblasts, the fatty acid uptake in the combined treatment of DEX and GW6471 was significantly lower than that in DEX treatment only (*P* < 0.05, Figure 5B). However, in OA pre-treated myoblasts, the fatty acid uptake in combined treatment of DEX and GW6471 had no changes compared with the DEX treatment only (*P* > 0.05, Figure 5B).

## Discussion

### FATP1 is involved in regulating skeletal muscle fat deposition in stressed broiler chickens

When the organism is suffers from stress, the GCs level rises, causing fat deposition in the mesenteric region of mice and reallocation of body energy (25–27). Studies on broiler chickens have proved that the GCs stress model could be established after injecting the DEX with 2 mg/kg of body weight into broiler chickens for 3–7 days (28–30), which was also used in the present study. Our present *in vivo* study showed that, after dexamethasone treatment of 2 mg per kilogram of weight for 3 days, the circulating INS and VLDL elevated, the fat deposition increased in abdominal tissue, liver and skeletal muscles, and the production performance impaired, indicating the establishment of the stress model where the glucolipid metabolism was compromised by GCs.

The performance results showed that the growth performance of broiler chickens decreased by DEX compared to pair-fed counterparts, indicating that GCs inhibited broilers growth even without considering the effect of GCs on feed intake (2, 3). However, GCs facilitated the fat deposition of chickens, which was demonstrated by the accumulation of abdominal fat, the increased TG content in muscle and liver tissues, and the increased blood VLDL level in the present study. These results were accordance with the previous studies showing that GCs would influence the fat metabolism and cause reallocation of fat (31–33).

By analyzing the expressions of fat metabolism-related genes in skeletal muscles, liver and abdominal fat, which were fat metabolically active tissues, we found that the FATP1 mRNA was promoted in muscle tissues but was inhibited in liver by DEX, indicating a tissue-specificity manner of FATP expression under stress. This implied that DEX might promote the uptake and deposition of skeletal muscle fat through the up-regulation of FATP1 transcription; in contrast, the effect of DEX on the hepatic fat deposition had little correlation with FATP1-mediated fat intake. Previous studies have shown that FATP1 is expressed the highest in the skeletal muscle tissues of broiler chickens, followed by the cardiac muscle tissues (34–38). These findings together confirmed the involvement of FATP1 in the fat transport and fat deposition of skeletal muscles under stress.

We further investigated the mechanism underlying the FATP1 regulation by GCs. According to the studies on humans, the increased mRNA level of the glucocorticoid receptor (GR) in skeletal muscle tissues was closely related to Type-II diabetes and obesity (39). When GCs combined with GR, the produced compound could relocate to the cell nucleus to combine with PPARs, which activated PPAR transcription. Since the promoter of FATP1 contained PPAR binding sites, PPAR activation could significantly stimulate the FATP1 transcription (40). The present results showed that mRNA levels of GR, PPARα, and FATP1 in thigh muscles after DEX treatment increased significantly, while those of livers reduced significantly, implying that GCs might regulate the FATP1 of skeletal muscles and livers through the GR-PPARα pathway. Adiponectin is one of the main cytokines secreted by adipose cells and was discovered in the 1990s. It regulates the PPAR and AMPK pathways of muscles and livers through receptors, ADPNR1 and ADPNR2, to further regulate fat metabolism (41, 42). The more the adiponectin is secreted, the less fat deposition in the broiler chicken (43). We thus detected the expressions of adiponectin as well as its receptors and found that DEX treatment resulted in a significant down-regulation of the adiponectin mRNA expression in abdominal fat tissues. Besides, the adiponectin receptor expressions were increased in the thigh muscles and breast muscles but decreased in livers by DEX. These implied that stress might affect fat metabolism by influencing adiponectin secretion and adiponectin receptor expression (44). Taken together, GCs may promote the fat uptake and deposition into skeletal muscle by up-regulating FATP1 transcription through GR-ADPNR-PPARα pathway.

### The FATP1 regulation by GCs was affected by fatty acid substrate

Studies have shown that unsaturated fatty acids play a critical role in preventing Type-II diabetes and obesity (45). However, how saturated and unsaturated fatty acids affect the fat deposition of chicken's myoblasts has not been confirmed yet (20). In the present study *in vitro*, compared to control, DEX did not affect the myoblast fat deposition and PPARα and FATP1 expressions without the external fatty acid, implying that DEX regulation on fatty acid uptake and fat deposition required for the fatty acid substrate. Under PA pre-treatment, both myoblast fatty acid uptake and fat deposition were promoted by DEX treatment; while under OA pre-treatment, the myoblast fat deposition was not affected by DEX, and the fatty acid uptake was decreased by DEX at 500 nM compared to control. These results indicating that the effect of GCs on myoblast fat uptake and deposition was related to the type of fatty acids substrate - saturated fatty acids were favorable for fat uptake and deposition, while unsaturated fatty acids were not.

Similarly, the regulation of GR-ADPNR-PPARα-FATP1 pathway by GCs was dependent on fatty acid type in the present study. In OA pre-treated myoblasts, DEX exerted almost ignorable effect on the gene expressions of *ADPNR, PPAR*α, and *FATP1*, indicating that the effect of GCs on unsaturated fatty acid uptake might not be through GR-ADPNR-PPARα-FATP1 pathway. However, for PA pre-treated myoblasts, the effects of DEX on the gene expressions of *GR, ADPNR, PPAR*α, and *FATP1* were identical, all of which upregulated first and then downregulated as the dose of DEX increases. These finding was not consistent with the previous research which showed that the FATP1 gene expression was affected independent of the type of fatty acids (46), we thus speculate that the dependence of FATP on fatty acids only occurs under stress.

We further confirmed the roles of GR and PPARα in GCs regulation under different fatty acid substrates by using their specific inhibitors, and we found again that DEX respectively decreased and increased the fatty acid uptake under OA pre-treatment and PA pre-treatment. When GR and PPARα were inhibited, the effects of DEX on fatty acid uptake were reversed for PA pre-treated myoblasts but not for OA pre-treated myoblasts. These results further confirmed that GCs could not affect the uptake of unsaturated fatty acids through the GR-PPARα pathway, while GCs enhanced the uptake of saturated fatty acids through the GR-PPARα pathway. GR-PPARα pathway could serve as potential targets for alleviating ectopic fat deposition in skeletal muscle of broilers caused by stress.

## Conclusions

Fatty acid transport protein 1 is involved in regulating skeletal muscle fat deposition in stressed broiler chickens which are induced by GCs. The FATP1 regulation by GCs was affected by fatty acid substrate - saturated fatty acids were favorable for fat uptake and deposition, while unsaturated fatty acids were not. GCs may affect the ADPNR-PPARα-FATP1 pathway by binding to its receptors, thus regulating the uptake of saturated fatty acids into myoblasts.

## Data availability statement

The original contributions presented in the study are included in the article/supplementary files, further inquiries can be directed to the corresponding author/s.

## Ethics statement

The animal study was reviewed and approved by Institutional Animal Care and Use Committee of Shandong Agricultural University.

## Author contributions

MW performed the experiments and wrote the manuscript. XW conceived the project and designed the protocol. All authors read and approved the final manuscript.

## Funding

This work was supported by the National Key Research and Development Program of China [grant number 2021YFD1300405] and the National Natural Science Foundation of China [grant number 31301993 and 31672441].

## Conflict of interest

The authors declare that the research was conducted in the absence of any commercial or financial relationships that could be construed as a potential conflict of interest.

## Publisher's note

All claims expressed in this article are solely those of the authors and do not necessarily represent those of their affiliated organizations, or those of the publisher, the editors and the reviewers. Any product that may be evaluated in this article, or claim that may be made by its manufacturer, is not guaranteed or endorsed by the publisher.
